# Non-Invasive Prostate Cancer Characterization with Diffusion-Weighted MRI: Insight from *In silico* Studies of a Transgenic Mouse Model

**DOI:** 10.3389/fonc.2017.00290

**Published:** 2017-12-01

**Authors:** Deborah K. Hill, Andreas Heindl, Konstantinos Zormpas-Petridis, David J. Collins, Leslie R. Euceda, Daniel N. Rodrigues, Siver A. Moestue, Yann Jamin, Dow-Mu Koh, Yinyin Yuan, Tone F. Bathen, Martin O. Leach, Matthew D. Blackledge

**Affiliations:** ^1^Department of Circulation and Medical Imaging, Norwegian University of Science and Technology (NTNU), Trondheim, Norway; ^2^St. Olavs University Hospital, Trondheim, Norway; ^3^Division of Molecular Pathology, Centre for Evolution and Cancer, Centre for Molecular Pathology, The Institute of Cancer Research, London, United Kingdom; ^4^CRUK Cancer Imaging Centre, Division of Radiotherapy and Imaging, The Institute of Cancer Research and Royal Marsden NHS Foundation Trust, London, United Kingdom; ^5^Prostate Cancer Targeted Therapy Group, Drug Development Unit, The Institute of Cancer Research and Royal Marsden NHS Foundation Trust, London, United Kingdom; ^6^Department of Pharmacy, Nord University, Namsos, Norway; ^7^Department of Laboratory Medicine, Women’s and Children’s Health, Norwegian University of Science and Technology (NTNU), Trondheim, Norway

**Keywords:** diffusion-weighted imaging, cellularity, whole-slide histology, mouse models of cancer, prostate cancer

## Abstract

Diffusion-weighted magnetic resonance imaging (DWI) enables non-invasive, quantitative staging of prostate cancer via measurement of the apparent diffusion coefficient (ADC) of water within tissues. In cancer, more advanced disease is often characterized by higher cellular density (cellularity), which is generally accepted to correspond to a lower measured ADC. A quantitative relationship between tissue structure and *in vivo* measurements of ADC has yet to be determined for prostate cancer. In this study, we establish a theoretical framework for relating ADC measurements with tissue cellularity and the proportion of space occupied by prostate lumina, both of which are estimated through automatic image processing of whole-slide digital histology samples taken from a cohort of six healthy mice and nine transgenic adenocarcinoma of the mouse prostate (TRAMP) mice. We demonstrate that a significant inverse relationship exists between ADC and tissue cellularity that is well characterized by our model, and that a decrease of the luminal space within the prostate is associated with a decrease in ADC and more aggressive tumor subtype. The parameters estimated from our model in this mouse cohort predict the diffusion coefficient of water within the prostate-tissue to be 2.18 × 10^−3^ mm^2^/s (95% CI: 1.90, 2.55). This value is significantly lower than the diffusion coefficient of free water at body temperature suggesting that the presence of organelles and macromolecules within tissues can drastically hinder the random motion of water molecules within prostate tissue. We validate the assumptions made by our model using novel *in silico* analysis of whole-slide histology to provide the simulated ADC (sADC); this is demonstrated to have a significant positive correlation with *in vivo* measured ADC (r^2^ = 0.55) in our mouse population. The estimation of the structural properties of prostate tissue is vital for predicting and staging cancer aggressiveness, but prostate tissue biopsies are painful, invasive, and are prone to complications such as sepsis. The developments made in this study provide the possibility of estimating the structural properties of prostate tissue via non-invasive virtual biopsies from MRI, minimizing the need for multiple tissue biopsies and allowing sequential measurements to be made for prostate cancer monitoring.

## 1 Introduction

Affecting one in eight during their lifetime, prostate cancer (PCa) remains the most common cause of cancer in men (1). Although patient prognosis has continued to improve, 5-year relative survival is strongly correlated with cancer stage at diagnosis (1). A recent European-wide study has demonstrated preliminary evidence that prostate cancer screening can improve outcomes for patients and prolong life where cancer is detected at an earlier stage (2). This provides options for radical treatment such as radiotherapy or prostatectomy, or non-interventional strategies such as active surveillance and watchful waiting (3). The usefulness of PCa screening, however, remains controversial; the most common screening tool, measurement of prostate-specific antigen (PSA) serum levels, is prone to overdiagnosis and overtreatment in up to 50% of cases (2, 4–7). PSA levels can be high in patients due to confounding processes including chronic prostatitis, benign prostatic hyperplasia, sexual activity, and old age (8), causing false positive test results in 76% of men with an elevated PSA level (9). Current guidelines in the United Kingdom, therefore, suggest that PSA measurements should only be performed in men following informed patient consent and understanding of the associated risks (10).

A recent study has shown that multiparametric magnetic resonance imaging (mpMRI) offers high sensitivity for detecting prostate cancer and could be used to triage men for prostate biopsy (11). Furthermore, MRI-targeted biopsies may minimize the number of biopsies required from each patient and reduce the risk of sepsis and other biopsy-related side effects (12). Reporting of mpMRI has recently been adopted into clinical practice for PCa detection and demonstrated utility in the assessment of local disease recurrence following radical treatments (13–20). Of particular importance is diffusion-weighted MRI (DWI) (14), which offers *in vivo* measurement of the apparent diffusion coefficient (ADC) to quantitatively identify prostate cancer (21): lower ADC values appear to be associated with more biologically aggressive cancers, providing a promising biomarker that is likely to impact on active surveillance programmes (21–25). Unfortunately, the biolophysical processes leading to differences in measured ADC values are poorly understood. It is widely hypothesized that ADC provides a surrogate marker of tissue cellularity (the number of cells per unit volume) (26), but in the prostate, this simplistic association does not account for the glandular structure of the prostate, which is likely to have great impact on the measurement of ADC *in vivo* (27–29).

In this study, we investigated the relationship between MRI-derived ADC and the structural properties of prostate tissue derived from whole-slide histology analysis of the transgenic adenocarcinoma of the mouse prostate model (TRAMP). This model recapitulates the progression of PCa observed in human disease, with initial onset characterized by prostatic intraepithelial neoplasia (PIN), leading to well-differentiated (WD) and then poorly-differentiated (PD) disease (30–33). It has been recently demonstrated that ADC can discriminate between these tissue types (34). By quantifying the relationship between ADC, cellularity, and the fraction of space occupied by prostate lumina (the luminal fraction), we aim to demonstrate the broader utility of ADC to inform on tissue characteristics of the entire prostate. In addition, we introduce the simulated-ADC (sADC) map, an *in silico* surrogate for *in vivo* ADC measurements calculated through computer simulation of the motion of diffusing water molecules within whole-slide histology images. The sADC provides a unique opportunity to compare the diffusion characteristics probed by MRI with parameters derived from histology for each pixel and at high resolution.

## 2 Theory

A model of MR-measured Apparent Diffusion Coefficient (ADC) building on histological estimates of tissue cellularity was derived using the principles of stereology (35). We model the MR imaging voxel to consist of two compartments: (i) an arrangement of volumes within which no water diffusion occurs (one volume per cell) bathed in (ii) a compartment of diffusing water. No water exchange is permitted between these compartments.

**Figure 1:**
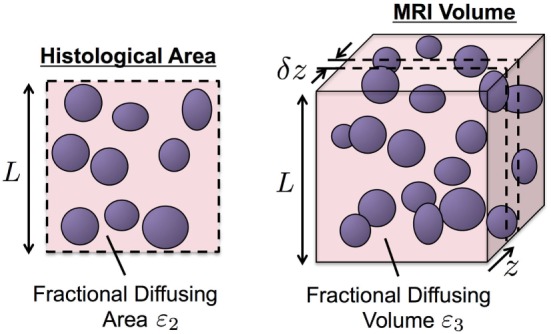
An illustration of our biophysical model for water diffusion within prostate tissue. Purple regions are impermeable to water diffusion that occurs in the surrounding space. Histology provides higher resolution than MRI but is limited to two-dimensional cross-sections of the tissue of interest (left). The principle of stereology provides approximation of volume fraction diffusing fluid, *ε*_3_, from its cross-sectional area fraction, *ε*_2_, estimated from histology samples (*ε*_3_ ≈ *ε*_2_).

A histology section is considered to be a finitely thin cross-section of the MR imaging voxel with thickness δ*z* at position *z*, in our case, along the MRI slice-encode direction (Figure 1). Without loss of generality, we assume the MR imaging voxel and histological pixel to be isotropic length *L*. The fractional area of diffusing fluid within a histology pixel is given by:

(1) $$\varepsilon_{2}\left(z\right)=1-\bar{A_{n}}\cdot \text{C}\left(z\right)$$

where $\bar{A_{n}}$ represents the average cross-sectional area of non-diffusing volumes (assumed to be constant over the thickness of the MR-imaging voxel), and *C*(*z*) is the cellularity of the pixel (number of cells per unit area). We approximate the MR voxel as a piecewise summation of histological pixels in the *z*-direction such that the volume of diffusing fluid within an MR voxel may be approximated from its fractional area by:

(2) $$V_{f}=\overset{L}{\underset{0}{\int }}\;L^{2}\varepsilon_{2}\left(z\right)\text{d}z=L^{3}\cdot \left(1-\bar{A_{n}}\cdot \bar{\text{C}}\right)$$

where $\bar{\text{C}}=\frac{1}{L}\int_{0}^{L}\;\text{C}\left(z\right)\text{d}z$ represents the average cellularity across the imaging voxel thickness. In an ideal case, $\bar{\text{C}}$ could be measured from a number of histological slices acquired over the thickness of the imaging voxel, but here, we assume our measured cellularity within a single histology image to be a good approximate ($\bar{\text{C}}\approx \text{C}$). We, therefore, conclude that the fractional area of diffusing fluid derived from a cross-section of the MR voxel provides a good estimate for its volume fraction:

(3) $$\begin{array}{llll} \varepsilon_{3} & =\frac{V_{f}}{L^{3}}\; & & \;\\ & \approx 1-\bar{A_{n}}\cdot \text{C}=\varepsilon_{2} \end{array}$$

To relate ADC to cellularity, we use a previously described power-law (36, 37):

(4) $$\varepsilon_{3}^{\tau }=\sqrt{\frac{\text{ADC}}{{\text{D}}_{0}}}$$

where D_0_ is the diffusion coefficient of fluidic component and τ, the “tortuosity exponent,” represents the degree to which water mobility is impeded by the presence of the non-diffusing volumes, and is thought to depend on the structural shape of the medium through which diffusion occurs (37). We validate a two-dimensional version of this power-law through Monte-Carlo simulations based on our histological specimens (Appendix B in Supplementary Material).

Combining equations (3) and (4), we derive the following relationship between MR-measured ADC and histology-derived estimates of cellularity:

(5) $$\text{C}=\frac{1}{\bar{A_{n}}}\left(1-{\left(\frac{\text{ADC}}{{\text{D}}_{0}}\right)}^{\frac{1}{2\tau }}\right)$$

## 3 Materials and Methods

### 3.1 Cellularity Calculation

**Figure 2:**
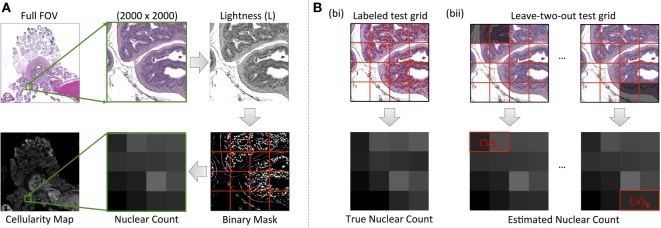
An illustration of our cellularity calculation methodology from HES images. **(A)** Demonstrates the workflow for the value of each pixel of the final cellularity map from subregions measuring 500 × 500 pixels in the HES slide (outlined by red lines). **(B)** Demonstrates our leave-two-out cross-validation process for optimizing the luminosity threshold used in cellularity calculations via the use of a manually labeled test grid [red circles labeling nuclei in **(B)**, bi]. The cross-validation score (CVS) was used as a means to determine the accuracy of the technique **(B)**, bii.

Maps of cellularity (estimated as number of nuclei per unit area) were calculated for each digitized slide according to the method presented in Figure 2A: HES images were initially converted from the RGB color space into the lightness channel (L) of the Lab color space. An optimum threshold, t_opt_, was then used to classify all pixels with L < t_opt_ as positively stained nuclei, thus creating a binary segmentation mask. The binary mask was subdivided into a regular grid of square subregions, each measuring 500 × 500 pixels (0.115 mm^2^ resolution), to represent the pixels in the final cellularity map. Within each subregion, the ratio of the total area covered by the mask to the mean nucleus cross-sectional area, provided an estimate of nuclear count (the mean ± SE cross-sectional area of nuclei was estimated to be 41.29 ± 0.42 µm^2^ from 1,110 manually contoured nuclei from representative HES histology images including all tissue types). The final cellularity pixel value was thus derived by taking the ratio of the estimated nucleus count to the total area in each subregion.

Our optimization strategy for determining segmentation thresholds is outlined in Figure 2B. A representative region was chosen from each digitized slide measuring 2,000 × 2,000 pixels (0.46 mm × 0.46 mm) and then subdivided into a regular grid of the same dimensions used in cellularity calculation described above. To provide a gold-standard cellularity estimate, a manual nucleus count was performed in each subregion (Figure 2B, i). Using 14 out of the 16 subregions, we then used a Levenberg–Marquardt algorithm to find the luminosity threshold that minimized the sum of absolute differences between the cellularity estimates for each subregion estimated using the above algorithm and the reference gold-standard (Figure 2B, ii). The remaining two subregions provided an unbiased estimate of the uncertainty of the threshold (the cross-validation score, CVS). This process was repeated eight times by leaving-out each combination of neighboring subregions but ensuring a subregion was not re-used. The final threshold used for the digitized slide was chosen as the mean result from cross-validation and the CVS provided an estimate on the uncertainty of the segmentation strategy (a median uncertainty of within 9.42% with a range of 0.98% for the fifth percentile to 42.16% for the 95th percentile across all samples).

### 3.2 Derivation of Luminal Fraction Maps from Histology

**Figure 3:**
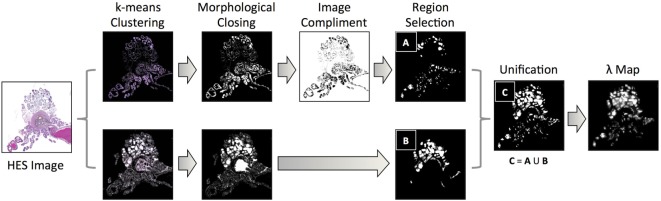
A flowchart of methodology for acquiring estimates of the fractional space occupied by lumina within histology images: k-means clustering of color channels in the LAB image color space derived six classes from HES images, which were converted to binary and processed using morphological closing and region labeling. For classes that identified the exterior of the lumina, the image-compliment was extracted; labeled areas above and below a threshold were discarded (region selection). Identified areas from selected clusters were unified and the fraction of pixels occupied by lumina within pixel-regions represented a single pixel in the final luminal fraction, λ-map.

Our methodology for extracting luminal fraction (λ) maps from histology is illustrated in Figure 3: images were converted from the RGB into the Lab color space and an unsupervised K-means clustering algorithm (k = 6) was applied on the color channels (A and B). A subset of the segmented clusters was manually selected and converted to binary. Morphological closing was performed using a small disk-shaped structuring element and spatially separate regions were identified using region labeling. Any regions whose size was deemed to be too small/large (e.g., due to background noise) were discarded. Each pixel of the λ-map was computed by calculating the fraction of pixels within each 20 × 20 pixel grid identified as luminal space in these images. This method was implemented in Matlab (Mathworks, Natick, MA, USA).

### 3.3 Simulation of Apparent Diffusion Coefficient from Histology

**Figure 4:**
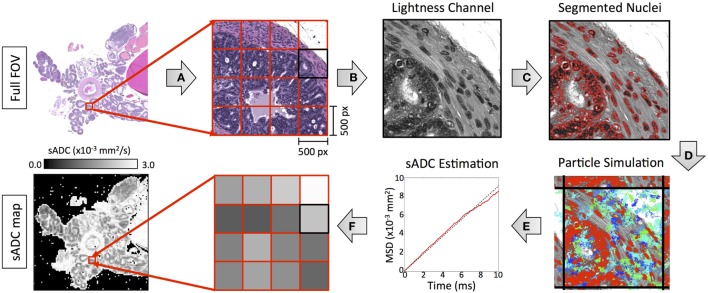
An illustration of our methodology for deriving simulated-ADC (sADC) maps from segmented HES images using the following steps: **(A)** The original HES image was divided into subregions measuring 500 × 500 pixels (outlined in red). **(B)** For each subregion, the luminosity channel was derived from the original RGB color space from which **(C)** nuclear boundaries were detected using our segmentation strategy (red outlines). **(D)** The trajectory of 10,000 diffusing particles was simulated within the freely diffusing space over 1,000 time increments, each lasting 10 µs such that the total diffusion time matched that of our MRI experiment (blue/green lines represent the trajectory of 100 of these particles). Where a boundary occurred, the subregion was tessellated to approximate an infinite spatial field. **(E)** The gradient of the particle mean-square-displacement curve, over the duration of the simulation time provided an estimate of the simulated-diffusion coefficient of the particles within the sub-region. **(F)** The sADC within each subregion provided a single pixel-value in the resulting sADC map.

We simulated the motion of water molecules within the diffusing space derived from histology samples according to the method outlined in Figure 4. Digitized images from HES slides were divided into subregions, each measuring 0.115 mm × 0.115 mm to represent a single pixel in the final sADC map (resolution matched to cellularity maps). Nuclei were segmented within each subregion using the strategy detailed in the preceding section, which provided a two-dimensional set, M(x,y) = M(x), of regions in which the particles were allowed to freely diffuse. The random trajectories of *N_p_* particles, with positions at time *t* denoted by $x_{i}^{t}\;\in \;R^{2}$, were simulated within the segmented diffusing space over *N_t_* time increments using the following algorithm:

**Table:** 

t = 0
Uniformly sample $x_{i}^{0}\in M$
**while** *t* < *N_t_* **do**
**for** *i* ∈ *N_p_* **do**
$\Delta x_{i}∼N\left(0,\sigma^{2}\right),\;\sigma^{2}=2D_{\text{f}}\frac{T}{N_{t}}$
**if** $x_{i}^{t}+\Delta x_{i}\in M$ **then**
$x_{i}^{t+1}\leftarrow x_{i}^{t}+\Delta x_{i}$
**else**
$x_{i}^{t+1}\leftarrow x_{i}^{t}$
**end if**
**end for**
*t* = *t* + 1
**end while**

$N\left(0,\sigma^{2}\right)$ represents a zero-centered Normal distribution with isotropic covariance *σ*^2^, *D*_f_ is the diffusion coefficient of free particles (set to 3.0 × 10^−3^ mm^2^/s to represent free water at 37°C) and *T* is the total time particles are allowed to diffuse. If a particle attempted to go past the edge of the image field-of-view, then the subregion was tessellated to approximate an infinite spatial field. The gradient of the mean-square-displacement curve of all particles over the duration of the simulation time provided an estimate of the simulated-diffusion coefficient of the particles within each sub-region. In our experiment, we used *N_p_* = 5,000 and *N_t_* = 2,630 (see Appendix A in Supplementary Material) with the total diffusion-time, *T* = 8.67 ms matched to that of our DW-MRI protocol.

### 3.4 Animals and Ethics

Animal care and experiments were carried out in accordance with Norwegian and EU guidelines for care and use of laboratory animals and were approved by the Norwegian National Animal Research Authority and the Norwegian Food Safety Authority (FOTS application 3823). The colony of TRAMP mice used as model organism in all animal experiments, were genetically modified from C57BL/6 mice (Jackson Labs, USA) and established in-house (NTNU, Norway). Genotyping was performed by PCR. The animals were kept in a standardized environment and monitored for general health status and body weight for the duration of the experiments. Male TRAMP (n = 9) and control (n = 6) mice from the same genetic background (C57BL/6) were imaged using MRI every 4 weeks from 8 weeks of age, and terminated at 28–30 weeks or when visual inspection of images indicated unacceptable tumor burden. Mouse body weights were recorded before each MRI session. In this study, results are presented from the final scanning time point only, where parameters from MRI and histology were compared.

### 3.5 Magnetic Resonance Imaging

MRI was performed on a 7T scanner (Biospec 70/20 Avance III, Bruker Biospin MRI, Ettlingen, Germany) with a volume resonator (86 mm diameter) for RF transmission and a phased array mouse heart surface coil for reception. Mice were anesthetized (≈2% isoflurane in medical air with 36% O_2_) for the duration of the MRI scan and positioned on the scanner bed in a prone position. Breathing motion in the pelvic region was reduced by firmly securing the mouse to the scanner bed with adhesive tape across its lower back. The respiration rate was monitored (SA Instruments, USA), and the body temperature was maintained at 37°C by circulating warm water through the bed. The following MR-imaging sequences were used: T2-weighted (T2W) images were acquired in the axial plane with an isotropic in-plane resolution of 0.1 mm and slice thickness of 0.33 mm using a RARE spin echo sequence (TE = 36 ms, TR = 5.5 s). Diffusion-weighted (DW-MRI) were acquired over the same region of the mouse as T2W images to allow for image registration; a fat-suppressed Stejskal–Tanner prepared multi-shot EPI sequence was used with the following parameters: TE = 28.5 ms; TR = 3 s; b-values = 0, 100, 200, 400, 800 s/mm^2^ acquired along three orthogonal directions; averages = 4; matrix size = 128 × 128; slice thickness = 0.9 mm; in-plane resolution 0.2 mm × 0.2 mm; number of EPI segments = 4. ADC maps were calculated in Matlab (MathWorks, Natick, MA, USA) by voxel-wise fitting of the signal (S) averaged over all gradient directions using a monoexponential model for all b-values according to:

$$S\left(b\right)=S\left(0\right)\cdot e^{-b\cdot \text{ADC}}$$

where *S*(0) is the signal intensity where *b* = 0 s/mm^2^. Regions of interest (ROIs) were drawn by DKH on ADC maps around regions corresponding to those drawn on histology. T2W and high-resolution HES images were consulted to ensure corresponding regions were chosen on ADC and cellularity maps.

### 3.6 Histology and Slide Digitization

Upon sacrifice, the genitourinary (GU) tract (prostate, seminal vesicles, emptied bladder) was excised, weighed, and fixed in formalin (10%) for at least 48 h. Samples were embedded such that the sectioning plane was aligned with the MRI images, as described in Ref. (34), and MR-images were used to identify cancerous areas from which histology samples were acquired. Formalin-fixed paraffin embedded samples were sectioned (4 µm slice thickness) and stained with hematoxylin (ChemiTeknik AS, Norway), erythrosine B (Sigma-Aldrich, Norway), and saffron (ChemiTeknik AS, Norway) using an automatic slide stainer (Sakura Tissue-Tek^©^ Prisma™). Whole HES-stained slides were digitized into jpeg format using a Hamamatsu NanoZoomer XR (Hamamatsu, Japan) scanner (40× magnification) such that each pixel in the high-resolution digitized image had a square resolution of 0.23 µm. Anatomopathological regions of interest were highlighted on digital slides by a pathologist (Daniel N. Rodrigues) using the tools provided by NanoZoomer Digital Pathology (Hamamatsu, Japan). Benign areas were defined for analysis on basis of predominant tissue type and included: fat, smooth muscle, and benign glands. As the distinction between intra-glandular overgrowth of luminal cells, i.e., prostatic intra-epithelial neoplasm (PIN), and invasive gland-forming malignancies, i.e., adenocarcinomas, occasionally depends on markers that highlight micro-anatomical boundaries, PIN/adenocarcinoma was considered as a single category. Overtly invasive tumors with a solid growth pattern and complete absence of gland formation were defined as poorly differentiated carcinomas.

## 4 Results

**Figure 5:**
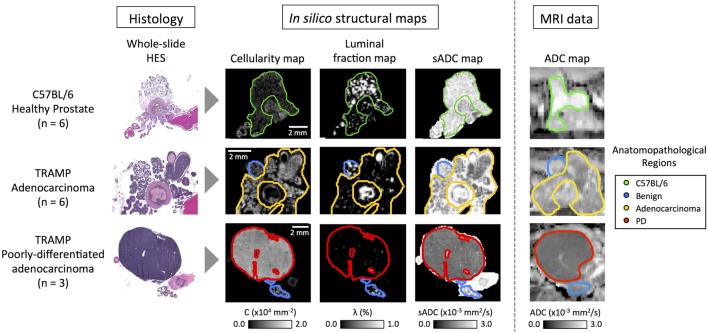
Representative images from C57BL/6, early stage cancer and advanced cancer TRAMP mice. Histology images were visually registered to diffusion-weighted MR-images and corresponding regions of interest were drawn on both modalities. Cellularity and simulated-ADC (sADC) maps were derived from histology images, while ADC maps were derived from MRI. Good registration was achieved and good correspondence between the histology derived sADC and *in vivo* measured ADC was observed. PD, poorly differentiated adenocarcinoma.

**Figure 6:**
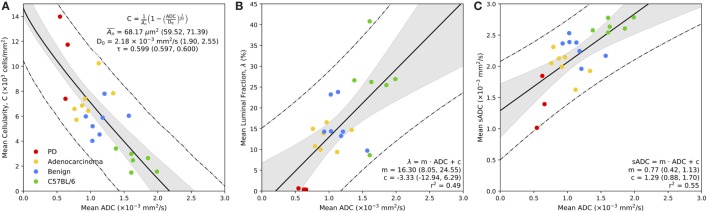
Correspondence between MR-derived ADC with mean cellularity **(A)**, mean luminal fraction **(B)**, and mean simulated sADC **(C)** estimated from whole-slide histology. Bold line represents the line of best fit of the model, as defined in the legend, the gray regions represent the 95% confidence intervals for the line of best fit, and the dashed lines represent the 95% prediction confidence. Parameters from model fitting are provided in the figure legend, with 95% confidence interval in parentheses.

Novel image analysis of digitized, whole-slide hematoxylin-eosin-saffron (HES)-stained histology slides were used to generate three quantitative maps of the microstructural properties in our mouse prostate tissue samples: (i) tissue cellularity, (ii) luminal fraction, and (iii) simulated-ADC (sADC). Figure 5 demonstrates examples of these maps from three of our samples; two from TRAMP specimens and one from a healthy control (C57BL/6). Good visual alignment between *in vivo* MRI and histology maps was achieved by matching anatomical landmarks such as the urethra and the different lobes of the mouse prostate. This allowed manual delineation of anatomopathological regions of interest (ROIs) by an experienced pathologist on (i) healthy prostate (from C57BL/6 mice), (ii) benign prostate (in TRAMP mice), (iii) adenocarcinoma, and (iv) poorly differentiated disease; corresponding ROIs were subsequently defined on MRI-derived ADC maps. Average values within these ROIs provided significant correlations between these histology-derived structural maps and the MRI-derived ADC maps as illustrated in Figure 6. Our model-fitting strategy is presented in Appendix B in Supplementary Material.

### 4.1 Cellularity versus MR-Derived ADC

Figure 6A demonstrates the relationship between mean estimates of MR-derived ADC and histology-derived cellularity (in units of 10^3^ cells/mm^2^) within the regions of interest defined on both modalities. There is a significant inverse relationship between ADC and tissue cellularity that is well described by our model (equation (5)); we estimate the diffusion coefficient of the fluidic compartment of our model to be D_0_ = 2.18 × 10^−3^ mm^2^/s (95% CI: 1.90, 2.55), the average cross-sectional area of the non-diffusing compartment to be $\bar{A_{n}}=68.17$ µm^2^ (59.52, 71.39) and the tortuosity exponent to be *τ* = 0.599 (0.597, 0.600). In addition to our model fitting approach, we performed a Leave-One-Animal-Out (LOAO) cross validation of the data that revealed this model was able to predict cellularity based on MR-derived ADC estimates to within a median deviation of 34.5% (normalized-root-mean-square-error = 0.18). This indicates that in this particular animal model, longitudinal measurements of ADC could provide a useful surrogate measurement of cellularity for monitoring disease progression and/or treatment response. We also observe a clear separation between normal and cancerous prostate tissue by both parameters, and ROI clustering of different cancer grades, providing evidence that ADC and cellularity serve as biomarkers for disease aggressiveness in prostate cancer.

### 4.2 Fractional Luminal Space versus MR-derived ADC

Figure 6B illustrates the significant positive correlation (r^2^ = 0.49) observed between average ADC and fractional space occupied by prostate lumina within histology (as a percentage). A linear fit to the slope provided a gradient of 16.3 (95% CI: 8.05, 24.55) with an intercept of −3.33 (95% CI: −12.94, 6.29). LOAO cross-validation analysis revealed this model was able to predict luminal space based on MR-derived ADC estimates to within a median deviation of 29.9% (normalized-root-mean-square-error = 0.20). This demonstrates that ADC may also provide a suitable biomarker for monitoring changes occurring to the glandular structure of the prostate during the onset of disease and/or during treatment. Luminal fraction also provided clear separation between poorly differentiated and well differentiated adenocarcinoma, indicating its utility for assessing tumor grade in the TRAMP model.

### 4.3 *In silico* Histology-Derived sADC versus MR-Derived ADC

A plot of the average MR-derived ADC against sADC revealed a significant linear trend (r^2^ = 0.55) between both parameters (Figure 6C). Linear fitting provided a gradient of 0.77 (95% CI: 0.42, 1.13) and intercept 1.29 (95% CI: 0.88, 1.70) indicating a positive bias in sADC compared to conventional ADC measurements. We attribute this bias to be a consequence of the assumption of free diffusion (diffusion coefficient of 3.0 × 10^−3^ mm^2^/s for free water at 37^∘^C) when simulating diffusion in histology images, ignoring the presence of any cell membranes, organelles, and/or macromolecules in the extracellular space. These data demonstrate that sADC may serve as a useful surrogate for MR-derived ADC when performing pixel-wise analysis of quantitative histology maps; spatial registration between MRI and histology is known to be problematic owing to a decrease in the size of histopathologic specimens following fixation and slide preparation.

### 4.4 Pixel-Wise Relationships between Structural Histology Metrics

**Figure 7:**
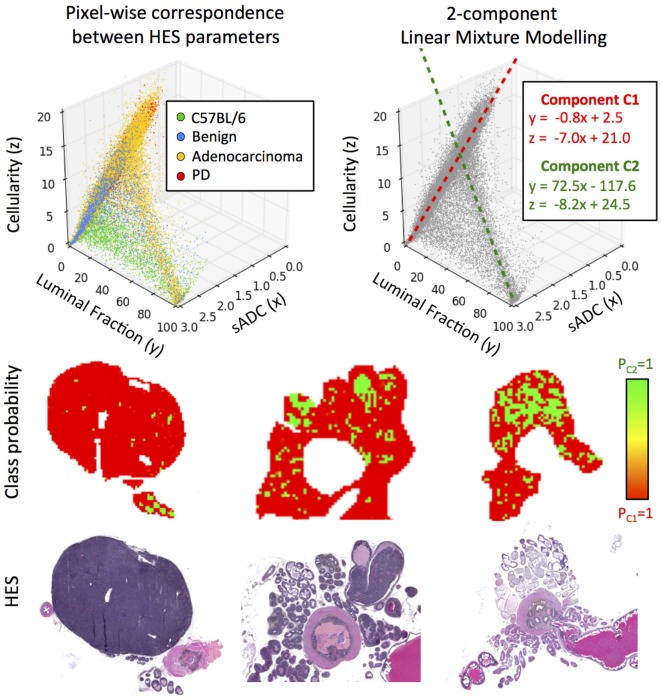
Top-left: scatter-plot of sADC (in units of ×10^−3^ mm^2^/s) versus luminal fraction (%) and cellularity (×10^−3^ cells/mm^2^). Top-right: two components are revealed through linear mixture modeling of these data; C1 and C2. Bottom: maps of the *a posteriori* class probability of histology reveals the spatial distribution of these compartments: C1 originating from luminal epithelium, smooth muscle and stroma, and C2 originating from prostate lumina.

Using sADC as a surrogate for *in vivo* MR-measurements of ADC allowed us to consider pixel-wise relationships between all three histologically derived parameters of prostate tissue morphology. Scatter-plots of these parameters (Figure 7) suggest that there are two principal linear compartments in these data; by performing a 2-component linear mixture model (LMM), we derived maps of the probability of each pixel on histology belonging to each of these two compartments. One compartment (C1) was observed to correspond to luminal epithelium, smooth muscle, and stroma, while the other corresponded to the presence of luminal space (C2). The linear relationships between sADC and cellularity in both of these compartments were revealed to be similar (linear gradient of −7.0 × 10^−6^ s/mm^4^ for C1 versus –8.2 × 10^−6^ s/mm^4^ for C2).

## 5 Discussion

In this study, we present novel techniques to probe the microstructural properties of healthy and diseased prostate from whole-slide histology samples of a transgenic mouse model of prostate cancer. This provides interpretation of the biological phenomena occurring at a microscopic level that determine the apparent diffusion coefficient measured at much larger length scales. Using these methods, we provide direct evidence for a significant inverse correlation between ADC and tissue cellularity.

We propose a compartmental model to relate MRI-derived ADC with prostate tissue cellularity measured on whole-slide histology. Our model consists of a non-diffusing compartment, which we liken to water-impeding boundaries such as the cell and nuclear walls, surrounded by a fluidic compartment, in which diffusion can occur. From our data, the model predicts an average area for the non-diffusing compartment of 68.17 µm^2^ (corresponding to a circular radius of 4.66 µm). Our proposed model further suggests that the relationship is non-linear, indicated by a tortuosity exponent that was estimated to be significantly different from 0.5 (p < 0.05). In addition, our model provides an estimate of the diffusion coefficient of the fluidic compartment to be lower than that of free water at 37^∘^C (3.0 × 10^−3^ mm^2^/s). We attribute this reduction in expected ADC to the presence of cell membranes, organelles in the cytoplasm, and macromolecules in the fluidic compartment, which act to hinder the motion of water.

We also observe a significant correlation between ADC and the proportion of space occupied by prostate lumina, indicating that the glandular structure of the prostate is an important consideration when ascribing biological interpretation to measured differences in ADC, as recently suggested in the context of T2-weighted MRI (38). This is also in accordance with previous findings in human prostate, where ADC was found to be strongly associated with the percentage distribution of luminal spaces (27).

Moving forward, current development in 3D-based histopathology with optical tomography (39), and fiducial markers to improve spatial co-registration of these modalities, will allow further developments to the findings presented in this study. The use of prostatectomy samples from human patients would further justify the clinical utility of these techniques, as the anatomical and histological appearance of TRAMP prostates differs from human tissues (40). Supportive patient studies should also include analysis at different clinical field-strengths (1.5 and 3.0T), where diffusion times may differ owing to the range of gradient-sets used. The range of values of ADC, cellularity, and luminal space reported in this study allowed us to differentiate cancer severity and construct quantitative models that predict cellularity and luminal space from ADC in this murine model of prostate cancer. Should similar results be found in the clinic, understanding these relationships may help clinicians interpret findings from *in vivo* ADC measurements in prostate cancer and relate them to the underlying tumor biology.

The gold standard for diagnosing prostate cancer is by Gleason grading of multi-core transrectal ultrasound-guided prostate (TRUS) biopsy (41). This invasive technique is prone to undersampling and underestimation of the grade (42) and does not lend itself to serial measurements on the same tissue. Preliminary evidence suggests that multi-parametric MR-guided biopsies improve prostate cancer detection over TRUS biopsy (43). Techniques such as the ones developed in this study provide evidence that MRI can be used as a non-invasive virtual biopsy to predict the location and severity of prostatic disease through targeted tissue sampling. This will spare patients from unnecessary invasive procedures and reduce the risk of biopsy related complications and disease misclassification.

## Ethics Statement

Animal care and experiments were carried out in accordance with Norwegian and EU guidelines for care and use of laboratory animals, and were approved by the Norwegian National Animal Research Authority and the Norwegian Food Safety Authority (FOTS application 3823). Animal care and experiments were carried out in accordance with Norwegian and EU guidelines for care and use of laboratory animals, and were approved by the Norwegian National Animal Research Authority and the Norwegian Food Safety Authority (FOTS application 3823).

## Author Contributions

DH, AH, KZ-P, DC, LE, DR, SM, YJ, DMK, YY, TB, ML, and MDB: substantial contributions to the conception or design of the work; or the acquisition, analysis, or interpretation of data for the work; drafting the work or revising it critically for important intellectual content; final approval of the version to be published; agreement to be accountable for all aspects of the work in ensuring that questions related to the accuracy or integrity of any part of the work are appropriately investigated and resolved.

## Conflict of Interest Statement

The authors declare that the research was conducted in the absence of any commercial or financial relationships that could be construed as a potential conflict of interest.
