# Supplementary material for: Non-Invasive Prostate Cancer Characterization with Diffusion-Weighted MRI: Insight from In silico Studies of a Transgenic Mouse Model
Source: Front Oncol. 2017 Dec 1;7:290. doi: 10.3389/fonc.2017.00290 (PMC5717839; doi:10.3389/fonc.2017.00290)
Supplement: Supplementary file 1 [file Data_Sheet_1.ZIP › Data Sheet 1/Appendix A.pdf]

# Appendix A. Optimising the number of steps and the number of particles for sADC calculations.

## Introduction

When computing estimates of the simulated apparent diffusion coefficient (sADC) from whole slide histology, there are two free parameters that must be chosen: (i) the number of particles used in the simulation, $\mathrm{N_p}$, and (ii) the number of steps used in the simulation, $\mathrm{N_s}$ (see Figure A1(a) for a visual representation of these parameters). In this appendix we describe how we chose both of these parameters for our study.

## Methods

A single patch consisting of 500×500 pixels was chosen from each whole-slide histology image (see Figure 3 of the full article for an example), and colour was converted into the lightness channel of the Lab colour space. Nuclei were segmented according to the methodology described in our paper. We simulated the trajectory of particles within the mask of regions defined to be outside the nuclei for a range of 22 values for $\mathrm{N_s} \in [10, 15000]$ and 15 values for $\mathrm{N_p} \in [2000, 10000]$. We repeated this process twice so as to calculate the standard deviation of sADC, $\sigma_{\mathrm{sADC}}$, as a function of $\mathrm{N_p}$ (experiments revealed no dependence of $\sigma_{\mathrm{sADC}}$ on $\mathrm{N_s}$).

## Results and Discussion

The dependence of sADC on $\mathrm{N_s}$ is demonstrated in Figure A1(b) for each 500×500 pixel image patch. It is clear that a bias is observed for low values of $\mathrm{N_s}$, which should be avoided for accurate sADC estimation. We chose $\mathrm{N_s}$=2630, as it provided a median bias (defined as the percentage difference from sADC calculated when $\mathrm{N_s}$=15000) of less than 1% across all samples. Figure A1(c) demonstrates how $\mathrm{N_p}$ does not affect the accuracy but only the precision of sADC estimation, $\sigma_{\mathrm{sADC}}$,. Fitting an inverse square-root law between $\sigma_{\mathrm{sADC}}$, and $\mathrm{N_p}$ provided a model between both parameters. We chose $\mathrm{N_p}$=5000 in our experiments to provide good SNR for sADC (49.4 for a true sADC of $1.5 \times 10^{-3}$ $\mathrm{mm^2/s}$) within a tolerable computation time. Using $\mathrm{N_s}$=2630 and $\mathrm{N_p}$=5000 resulted in a total computation time of approximately 3.22 seconds per 500×500 image patch on histology).

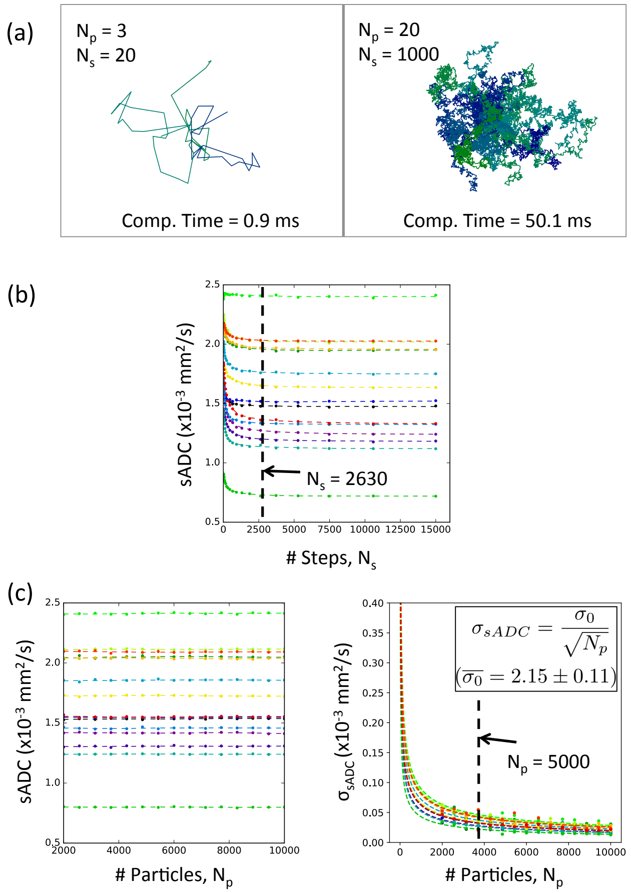


Figure A1: (a) Increasing the number of particles, $N_p$, and number of steps, $N_s$, used in the random walk for sADC estimation causes significant increase in computation time. (b) A value of $N_s$ causes loss in sADC accuracy where as a low Np causes a reduction in precision (c). Results for different animals are presented in different colours.
