# Supplementary material for: Non-Invasive Prostate Cancer Characterization with Diffusion-Weighted MRI: Insight from In silico Studies of a Transgenic Mouse Model
Source: Front Oncol. 2017 Dec 1;7:290. doi: 10.3389/fonc.2017.00290 (PMC5717839; doi:10.3389/fonc.2017.00290)
Supplement: Supplementary file 1 [file Data_Sheet_1.ZIP › Data Sheet 1/Appendix B.pdf]

Appendix B. Validation of Model Assumption and Data Fitting

Validation of model assumption

We validated a key assumption in our model (equation (4) of main article and reproduced in Figure B1) by performing a pixel-wise simulation of the diffusion within the extranuclear space of segmented histology images, sADC (simulated-ADC), and compared derived values with the estimated extranucelar area fraction, ε_2 (Figure B1). By fitting these data to equation (4) we derived a value for the tortuosity exponent, $\tau = 0.599$ (95% confidence interval: 0.597, 0.600), which is significantly different from a value that indicates linearity between pADC and ε_2 ($\tau = 0.5$). Furthermore, box-plots of data residuals from the derived model reveal no major discrepancies between different animals and/or tissue subtypes. This provides evidence for the assumption of a population-wide tortuosity component, τ, in our TRAMP and C57BL/6 mice.

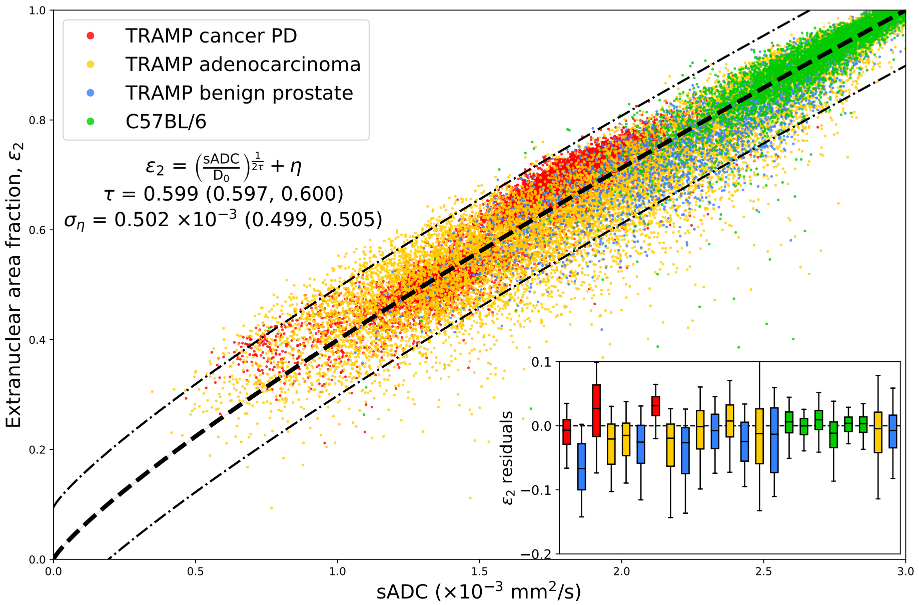

Figure B1: A plot of pixel-wise correspondence between histology derived estimates of simulated-ADC (sADC) against extranuclear area fraction ε_2 from all tissue samples. There is striking evidence that the diffusion model (bold dashed line) fits data from all tissue types and mice in our study. This is supported by a box-plot of the residuals for ε_2 (bottom-right) for each mouse and tissue-subtype combination. From this model we estimated the tortuosity exponent for this mouse population, τ (95% confidence intervals within parentheses). Model prediction confidence intervals (95%) are shown by the fine dashed line.

MCMC fitting

To fit this model, samples were drawn from the posterior probability distribution for the tortuosity component, n, using a Metropolis-Hastings algorithm [1] with a uniform prior distribution on $\tau > 0$. A total of 1000 samples were drawn from the posterior using 110,000 Markov-Chain Monte-Carlo (MCMC) simulations and discarding the first 10000 as burn-in and thinning the samples by 99% to ensure they were approximately independent (see Figure B2 for the MCMC convergence diagnostics). The *a-posterori* estimate of τ was derived as the median of the samples, and the 2.5 and 97.5 percentiles provided 95% confidence intervals. Moreover, posterior estimates of the variance of data residuals (using an uninformative inverse-gamma prior) were used to generate 95% prediction intervals.

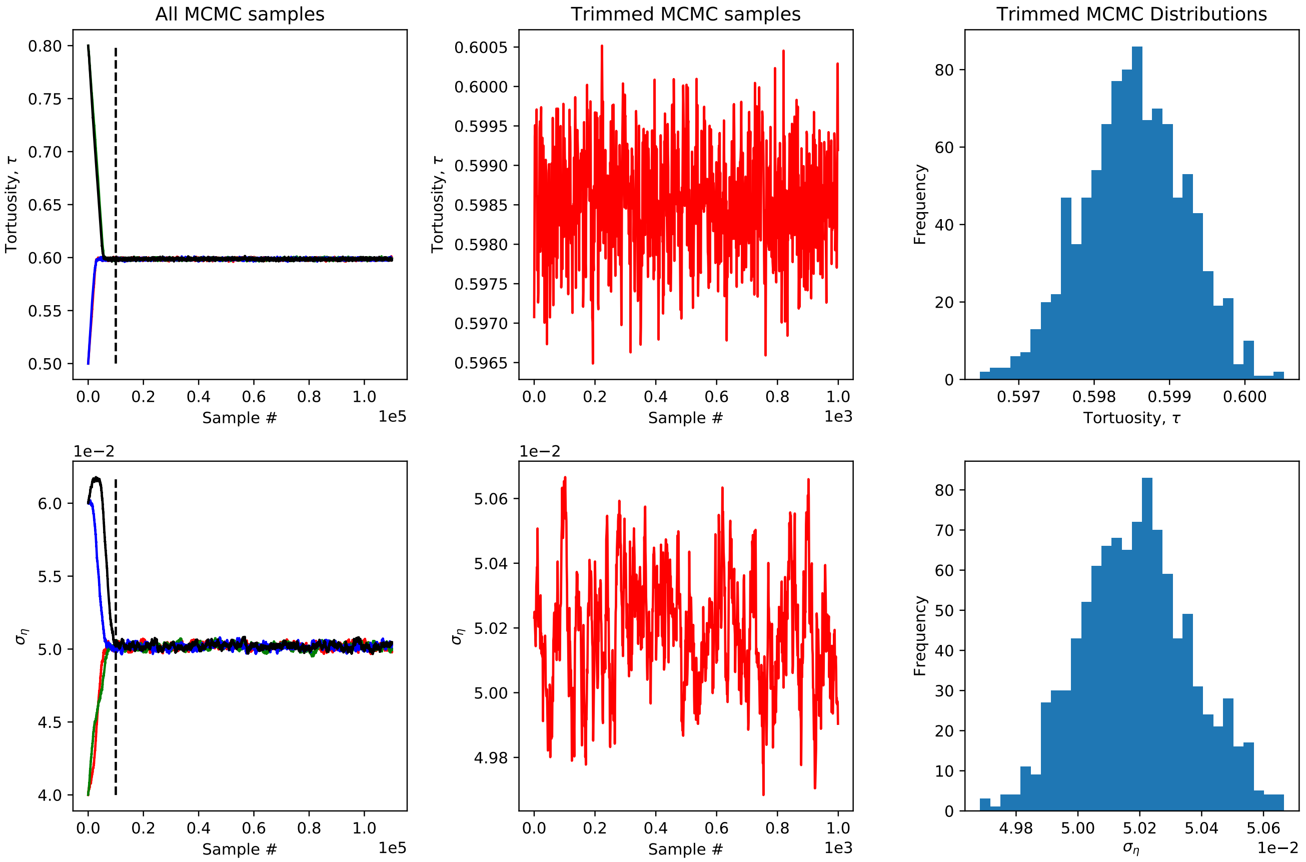

Figure B2: MCMC convergence diagnostics for model fitting between simulated-ADC (sADC) and extranuclear area fraction ε_2. Results for each parameter of the model are presented on each row. The trace of all generated MCMC samples are presented in the first column: starting the MCMC sampling from two different positions for each parameter (presented as different colours) provides intuition on the convergence of the sampling to a stable result. We chose to exclude the first 10000 samples as a burn-in period (indicated by dashed vertical line). The second column demonstrates a single MCMC trace for which the burn-in period has been removed, and the samples have been thinned by 99% to remove correlations between each sample. It is apparent that the samples are appropriately uncorrelated. The last column demonstrates the histograms of the trimmed MCMC samples.

MCMC fitting for our quantitative model

Our proposed model for relating histology-derived cellularity with MRI-derived ADC is (equation (5) of main article):

$$\mathrm{C} = \frac{1}{\overline{A}_n}\left(1 - \left(\frac{\mathrm{ADC}}{\mathrm{D}_0}\right)^{\frac{1}{2\tau}}\right) + \eta \quad (1)$$

where η has been introduced to represent noise in the model fit (assumed to be normally distributed: $\eta \sim \mathcal{N}(0, \sigma_\eta)$). Data were fit to this model by drawing samples from the posterior distribution of tortuosity exponent, τ, average cell area, $\overline{A}_n$, and extracellular diffusion coefficient D_0. The prior distribution for τ was derived from the posterior distribution for τ derived from the simulation experiment described above, whilst the prior distributions for the other parameters were uniform over specified ranges: $\overline{A}_n \in [0.0, 71.5]$ (µm^2) and $\mathrm{D}_0 \in [0.0, 5.0]$ ($\times 10^{-3}$ mm^2/s). A total of 1000 samples were drawn from the posterior using 110,000 Markov-Chain Monte-Carlo (MCMC) simulations and discarding the first 10000 as burn-in and thinning the samples by 99% to ensure they were approximately independent (see Figure B3 for the MCMC convergence diagnostics). The *a-posterori* estimate of all parameters was derived as the median of the samples, and the 2.5 and 97.5 percentiles provided 95% confidence intervals. Moreover, posterior estimates of the variance of data residuals (using an uninformative inverse-gamma prior) were used to generate 95% prediction intervals.

References

[1] W Keith Hastings. Monte carlo sampling methods using markov chains and their applications. *Biometrika*, 57(1):97–109, 1970.

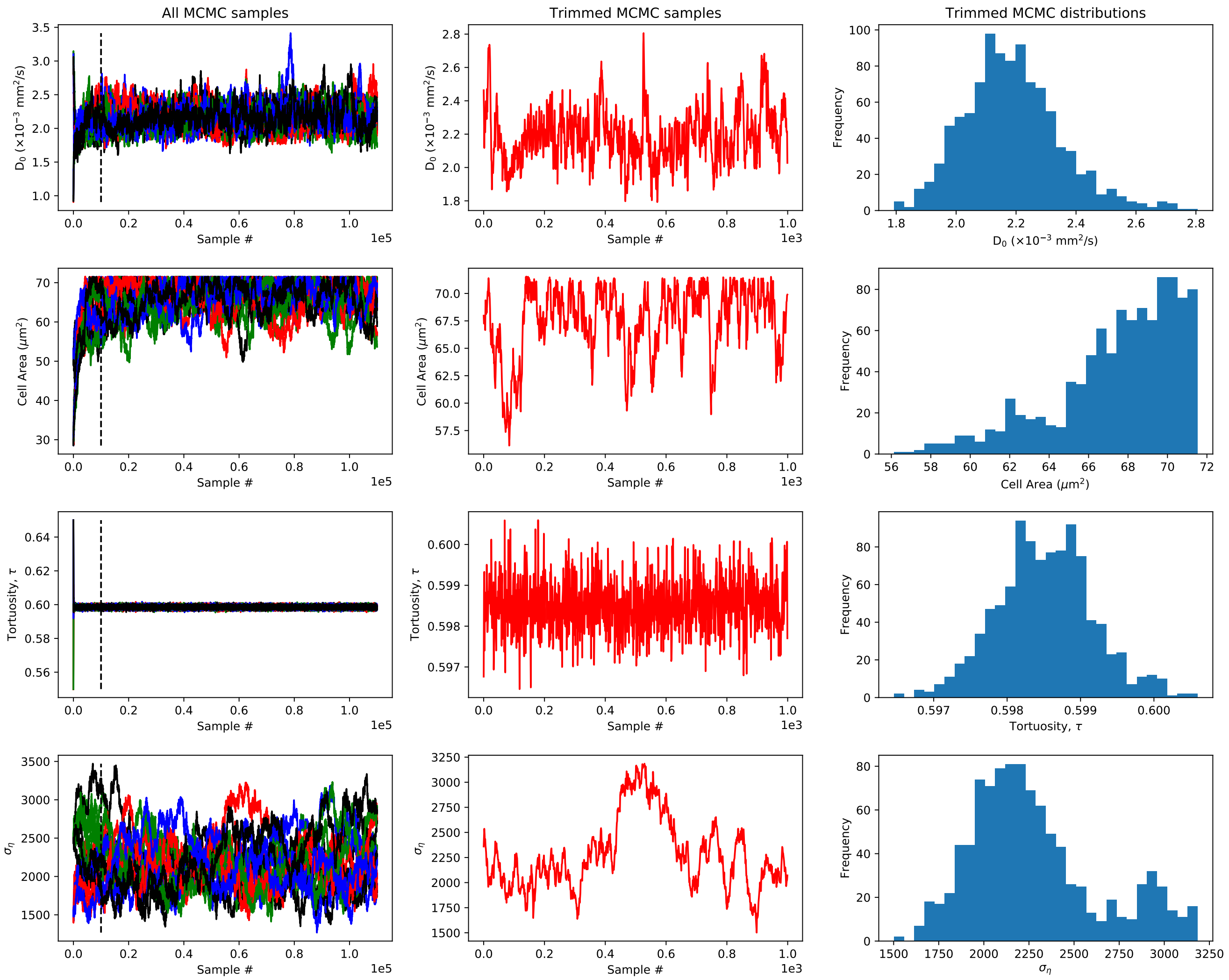

Figure B3: MCMC convergence diagnostics for model fitting between MRI-derived ADC and histology-derived cellularity (C). Results for each parameter of the model are presented on each row. The trace of all generated MCMC samples are presented in the first column: starting the MCMC sampling from two different positions for each parameter (presented as different colours) provides intuition on the convergence of the sampling to a stable result. We chose to exclude the first 10000 samples as a burn-in period (indicated by dashed vertical line). The second column demonstrates a single MCMC trace for which the burn-in period has been removed, and the samples have been thinned by 99% to remove correlations between each sample. It is apparent that the samples are appropriately uncorrelated. The last column demonstrates the histograms of the trimmed MCMC samples.
